# Medicinal Chemistry of *Annonaceous* Acetogenins: Design, Synthesis, and Biological Evaluation of Novel Analogues

**DOI:** 10.3390/molecules14093621

**Published:** 2009-09-17

**Authors:** Naoto Kojima, Tetsuaki Tanaka

**Affiliations:** Graduate School of Pharmaceutical Sciences, Osaka University, 1-6 Yamadaoka, Suita, Osaka, Japan

**Keywords:** *Annonaceous* acetogenins, antitumor activity, analogues, structure–activity relationship, polyketides

## Abstract

Most *Annonaceous* acetogenins are characterized by between one and three THF ring(s) with one or two flanking hydroxyl group(s) in the center of a C32/34 fatty acid, and the terminal carboxylic acid is combined with a 2-propanol unit to form an α,β-unsaturated γ-lactone. While many studies have addressed the properties and synthesis of natural acetogenins due to their attractive biological activities and unique structural features, a number of analogues have also been described. This review covers the design, synthesis, and biological evaluation of acetogenin analogues.

## 1. Introduction

**Figure 1:**
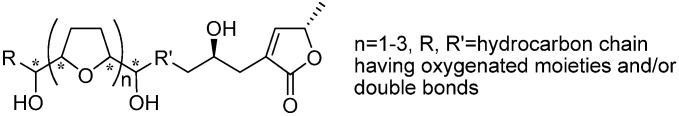
Representative structure of the *Annonaceous* acetogenins.

A new class of polyketides, the *Annonaceous* acetogenins, has been isolated from *Annonaceous* plants growing in tropical and subtropical regions. Since the isolation of the first acetogenin, uvaricin, more than 400 members of the family have been found and characterized (Figure 1) [1,2,3,4,5,6,7,8,9,10,11,12].

Most acetogenins are white waxy derivatives of long-chain fatty acids (C32 or C34), and the terminal carboxylic acid is combined with a 2-propanol unit at the C-2 position to form a methyl-substituted α,β-unsaturated-γ-lactone. One of their interesting structural features is a single, adjacent, or nonadjacent tetrahydrofuran (THF) or tetrahydropyran (THP) system with one or two flanking hydroxyl group(s) at the center of a long hydrocarbon chain. Biogenetically, it has been suggested that the THF or THP cores are generated by polyepoxidation of an unconjugated polyene followed by domino cyclizations.

In addition to their unique chemical structures, much attention has also been paid to acetogenins’ broad range of bioactivity; e.g., their immunosuppressive, antimalarial, insecticidal, antifeedant, and probably most important, antitumor activities. Some acetogenins show growth inhibitory activity against multidrug resistant (MDR) cancer cells [13]. It is generally accepted that the mode of action of acetogenins is the inhibition of NADH–ubiquinone oxidoreductase (complex I) in mitochondria [14]. Inhibition suppresses ATP production, especially for cancer cells with high metabolic levels, leading to apoptosis.

Several strategies for the total synthesis of natural acetogenins and their analogues have been reported, motivated by their unique structural features and attractive biological activities [15,16,17,18,19,20,21]. This review focuses on those analogues whose biological activities have been reported previously.

## 2. Modification of the Tetrahydrofuran Moiety

**Scheme 1:**
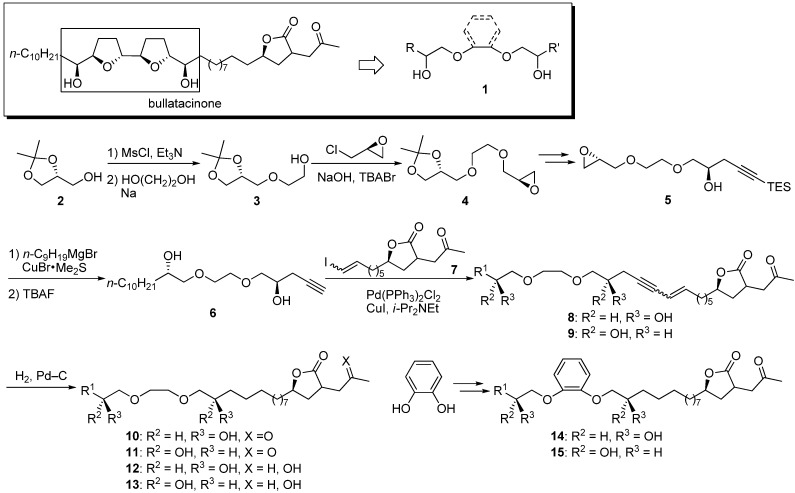
Synthesis of ethylene glycol and catechol analogues by Grée’s group.

Structural simplification of the tetrahydrofuran moiety, especially the bis-tetrahydrofuran group, is worthwhile because of the limited availability of these complex structures. Grée’s group reported the synthesis and biological activity of a series of acetogenin analogues **1** consisting of ethylene glycol and catechol ethers in place of the bis-tetrahydrofuran core of bullatacinone, which has a ketolactone moiety at the end [22,23,24]. Their analogues were designed to incorporate various lipophilic side chains in place of an *n*-alkyl chain. A representative synthetic pathway is given by the preparation of **10a** (Scheme 1). The synthesis of the β-hydroxyl ether core was achieved by condensation of the mesylate of solketal **2**, ethylene glycol, and epichlorohydrin. After opening of the epoxide with triethylsilylacetylide, the lipophilic side chain was introduced at the opposite end to give the alkyne **6**. Sonogashira coupling of **6** with the γ-lactone fragment **7**, followed by hydrogenation, afforded the target analogue **10a**. Twenty-three analogues were tested for cytotoxicity against L1210 leukemia cells (Table 1). Analogues containing catechol were more effective than the series of ethylene glycol derivatives. No significant differences were observed between the various lipophilic side chain substitutions. The simplified acetogenins showed less cytotoxic activity than the natural acetogenins annonacin, bullatacin, and bullatacinone. However, catechol derivatives showed an interesting effect on cell cycle. Natural acetogenins were equally cytotoxic to each phase of the cell cycle, but analogues **14a**, **14c**–**g**, and **15a** modified the cell cycle at either phase G1 or G2/M. The target pathway of these analogues may be different from the target of natural acetogenins.

**Table 1:** IC_50_ (μM) of synthetic analogues against L1210 cell lines.

R^1^	IC_50_	R^1^	IC_50_
*n*-C_10_H_21_ (**8a**)	3.5	*n*-C_10_H_21_ (**9a**)	1
Ph (**8b**)	19.8	Ph (**9b**)	21.6
*p*-MeOC_6_H_4_ (**8c**)	> 10	*p*-MeOC_6_H_4_ (**9c**)	32
*p*-CF_3_C_6_H_4_ (**8d**)	3.3	–	–
2-Nph (**13e**)	2.1	2-Nph (**9e**)	3
Bu_2_N (**8f**)	3.9	Bu_2_N (**9f**)	2.5
Oct_2_N (**8g**)	3.7	–	–
*N*-piperidinyl (**8h**)	28.7	*N*-piperidinyl (**13h**)	23.3
3-*O*-cholesteryl (**8i**)	2.8	3-*O*-cholesteryl (**13i**)	12.2
*n*-C_10_H_21_ (**14a**)	1.0	*n*-C_10_H_21_ (**15a**)	7.6
Ph (**14b**)	2.0	–	–
*p*-CF_3_C_6_H_4_ (**14d**)	2.2	doxorubicin	0.025
2-Nph (**14e**)	0.7	annonacin	0.042
Bu_2_N (**14f**)	1.3	bullatacin	0.0004
Oct_2_N (**14g**)	2.5	bullatacinone	0.016

**Scheme 2:**
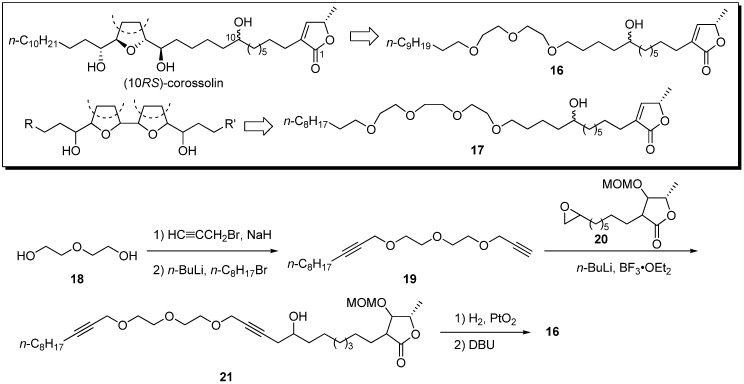
Synthesis of polyether analogues by Wu’s group.

In 2000, Wu’s group reported polyether mimics based on the ionophoric ability [25,26,27] of the THF moiety in acetogenins [28]. (10*RS*)-Corossolin was simplified to the diethylene glycol analogue **16**, and the corresponding bis-THF acetogenin, bullatin, was simplified to triethylene glycol **17**. A representative synthetic pathway is given by the preparation of **16** (Scheme 2). Bis-propargylation of diethyleneglycol **18** with propargyl bromide, followed by monoalkylation with *n*-octyl bromide, afforded the polyether **19**. The reaction of epoxide **20** with the acetylide of **19** yielded the alcohol **21**. Hydrogenation of triple bonds, followed by elimination of the MOM-oxy group, afforded analogue **16**. Preliminary screening showed that analogues **16** and **17** had moderate activity against HL-60 and K562 (Table 2).

**Table 2:** *In vitro* testing against the HL-60 and K562 cell lines.

**Conc. (μM)**	**IG%**					
	**for HL-60**			**for K562**		
	100	10	1	100	10	1
**16**	100	50	0	31	18	0
**17**	100	65	21	55	25	22
corossolone	68	29	0	53	16	2
(10*RS*)-corossolin	63	56	5	10	2	0
solamin	24	8	0	59	39	29
bullatacin	73	7	0	53	39	27

**Scheme 3:**
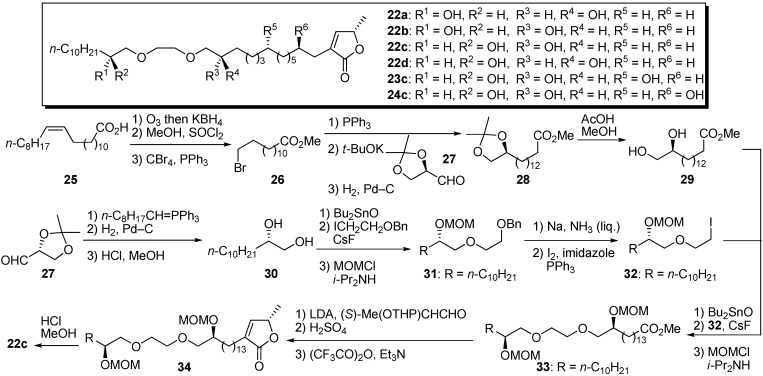
Synthesis of polyether analogues by Wu’s group.

Wu’s group reported the synthesis and biological activity of analogues similar to Gree’s, but Wu’s analogues had an α,β-unsaturated-γ-lactone at the end instead of the ketolactone of Gree’s derivatives [29,30]. They completed synthesis of the polyether analogues by a convergent strategy. A representative synthetic pathway is given by the preparation of **22c** (Scheme 3). The synthesis of fragment **29** began with the bromo ester **26** prepared from *cis*-erucic acid **25**. The Wittig olefination of the phosphonium salt prepared from **26** with (*R*)-glyceraldehyde acetonide **27** was followed by hydrogenation and removal of the acetonide protective group, yielding fragment **29**. The preparation of the other fragment **32** began with the chain extension of (*R*)-glyceraldehyde acetonide **27**. The resulting diol **30** was condensed with 2-benzyloxyethyl iodide via a cyclic stannate intermediate. After protection of the secondary alcohol, followed by deprotection of benzyl ether, the resulting primary hydroxyl group was converted to iodide to give **32**. The coupling reaction of fragments **29** and **32** was achieved by selective etherification with dibutyltin oxide and cesium fluoride. The γ-lactone moiety was introduced by way of the aldol strategy with *O*-THP-(*S*)-lactaldehyde. Deprotection of the MOM ether of **34** gave the polyether analogue **22c**. The synthesized samples were evaluated by MTT (3-(4,5-dimethylthiazol-2-yl)-2,5-diphenyltetrazolium bromide) assay to measure cytotoxicity against several human solid tumor cell lines (Table 3). All four samples showed potent activities against HCT-8 and HT-29 cell lines, whereas they had no cytotoxity against normal human cells. Although the (10*R*)-hydroxyl-substituted analogue **23c** showed activity similar to **22c**, the introduction of the (4*R*)-hydroxyl group into **24c** raised the potency by a factor of 15 [31]. It was found that most cell death induced by **22c** was due to necrosis, and **23c** affected mitochondrial complex I [32]. A preliminary antitumor assay in mice (Lewis lung cancer) with **22c** showed 60% inhibition of tumor compared with the control.

**Table 3:** Cytotoxicity against human solid tumor cell lines.

Compounds	EC_50_ [g/mL]			
	KB	A2780	HCT-8	HT-29
**22a**	> 1	> 1	6.6 × 10^−2^	2.72 × 10^−1^
**22b**	> 1	> 1	9.7 × 10^−2^	1.12
**22c**	> 1	> 1	3.2 × 10^−2^	1.1 × 10^−1^
**22d**	> 1	> 1	6.5 × 10^−2^	7.83
adriamycin	2.89 × 10^−3^	1.02 × 10^−3^	4.65 × 10^−3^	9.8 × 10^−4^

**Scheme 4:**
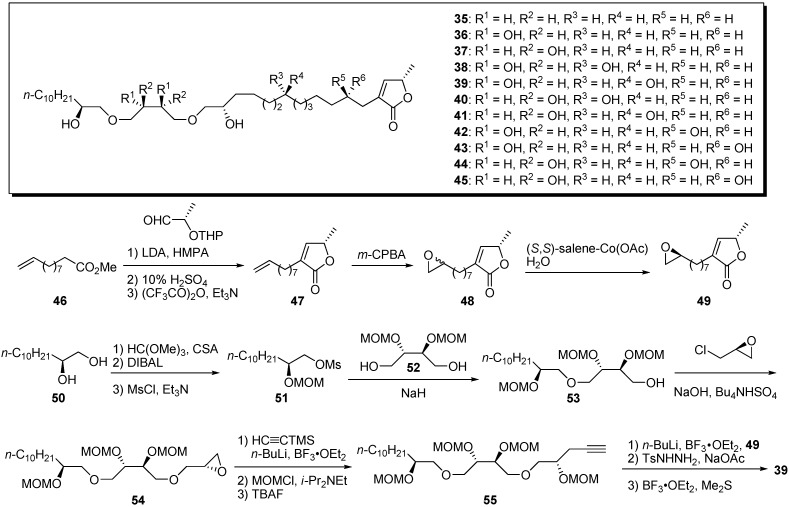
Synthesis by Yao and Wu’s group of polyether analogues with dihydroxyl groups in the vicinity of the ether bonds.

In 2004, Yao and Wu’s group reported preparation of a small library to clarify the structure–activity relationships (SAR) of their polyether analogues [33]. New analogues that had dihydroxyl groups in the vicinity of the ether bonds were prepared by convergent synthesis. A representative synthetic pathway is given by the preparation of **39** (Scheme 4). First, ester **46** was transformed to γ-lactone **47** by a three-step sequence involving an aldol reaction with *O*-THP-(*S*)-lactaldehyde. After epoxidation of the terminal olefin, Jacobsen’s hydrolytic kinetic resolution gave a chiral epoxide **49**. The preparation of the polyether fragment **55** began with the condensation of the mesylate **51** and tetraol derivative **52**. *O*-Alkylation of the alcohol **53** with (*R*)-epichlorohydrin afforded the epoxide **54**. Opening of the epoxide with trimethylsilylacetylide, followed by protection of the resulting secondary alcohol and deprotection of the TMS group, produced the polyether fragment **55**. The coupling reaction of the acetylide prepared from **55** and the epoxide **49**, followed by reduction of the triple bond and deprotection of the MOM group, yielded the polyether analogue **39**. Nearly all new analogues bearing hydroxyl groups in the vicinity of the ether bonds showed no activity against the Bel-7402 cell line, but exhibited good cytotoxicity against HT-29 and HCT-8 cell lines in the low micromolar range (Table 4). It is interesting that the introduction of hydroxyl group and their stereochemistry yielded selectivity among the tumor cell lines.

**Table 4:** Cytotoxicity against human solid tumor cell lines.

Compounds	IC_50_ [μΜ]			
	KB	Bel-7402	HT-29	HCT-8
**35**	7.65	1.99	0.099	0.11
**36**	4.02	> 10	1.84	3.49
**37**	13.13	> 10	5.72	8.58
**38**	13.81	> 10	7.19	5.71
**39**	23.30	> 10	9.79	10.00
**40**	9.68	> 10	4.56	24.46
**41**	21.30	> 10	7.22	6.14
**42**	6.75	> 10	3.60	3.39
**43**	6.38	> 10	2.36	3.51
**44**	2.00	> 10	1.75	2.00
**45**	2.35	4.14	1.51	3.46
adriamycin	< 0.01	0.95	0.055	0.11

**Scheme 5:**
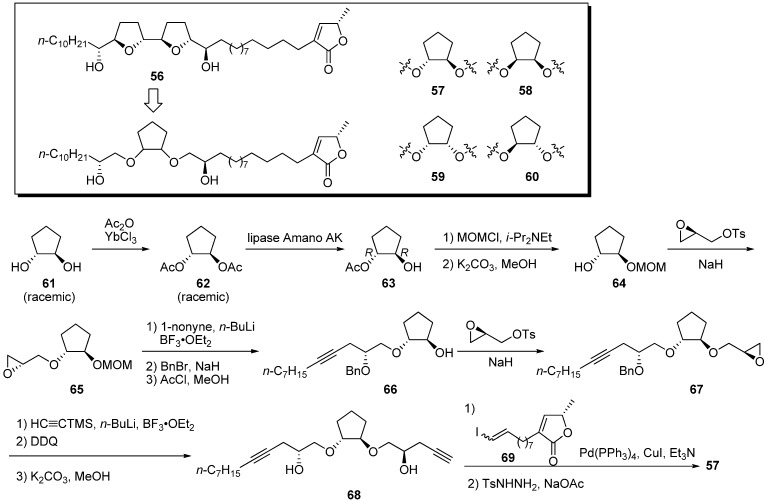
Synthesis of 1,2-cyclopentanediol bis-ether analogues by Miyoshi’s group.

Miyoshi *et al*. noted a structural similarity between the hydroxylated bis-THF moiety of natural acetogenins and the hydroxylated 1,2-cyclopentanediol bis-ether motif, especially the relative spatial positions of the four oxygen atoms (Scheme 5) [34]. Four diastereomeric 1,2-cyclopentanediol cores were synthesized by optical resolution with lipase. A representative synthetic pathway is given by the preparation of **57**. Acetylation of (±)-*trans*-1,2-cyclopentandiol **61**, followed by hydrolytic optical resolution, gave the chiral monoacetate **63**. After protection of the hydroxyl group, treatment with K_2_CO_3_ in MeOH afforded **64** in the enantiomerically pure form. Introduction of two remaining secondary hydroxyl groups was performed by a coupling reaction with (2*R*)-glycidyl tosylate followed by the ring opening of epoxide with acetylide. Sonogashira coupling of alkyne **68** and vinyl iodide **69**, followed by selective reduction of the triple bond and enyne, gave the target analogue **57**. Inhibitory activities of four mimics against bovine heart mitochondrial complex I were examined (Table 5). All analogues showed potent inhibition at the nanomolar level, being nearly equipotent with bullatacin, which is one of the most potent inhibitors of complex I. It was also shown that the stereochemistry of 1,2-cyclopentanediol bis-ether cores had a slight effect on inhibitory potency.

**Scheme 6:**
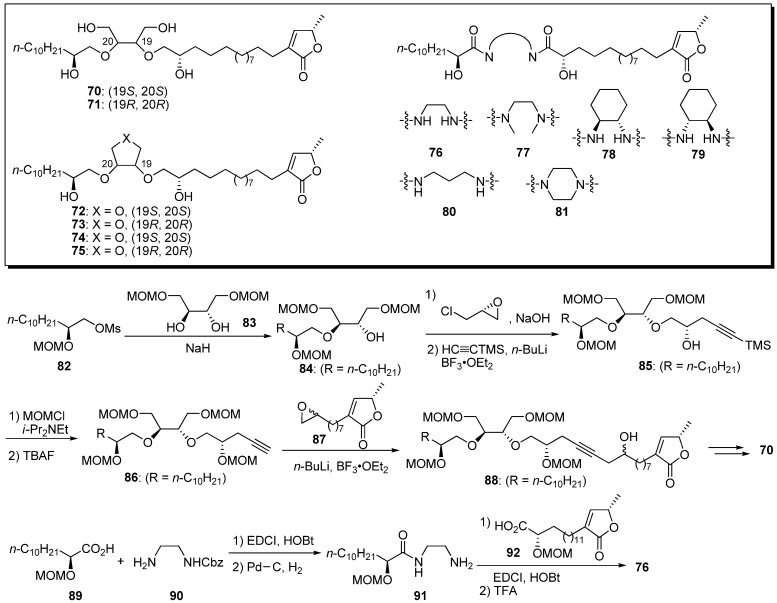
Synthesis of conformationally constrained polyether analogues by Yao’s group.

Yao *et al.* designed conformationally constrained analogues of the acyclic bis-ether mimics [35]. The 1,2-disubstitued ethylene glycol, tetrahydrofuran-3,4-diol, tetrahydrothiophene-3,4-diol, and bis-amide moieties were conformationally constrained to alter the ether functionality in the lead compound, **22c**. A representative synthetic pathway is given by the preparation of **70** and **76** (Scheme 6).Synthesis of the analogue **70** with a 1,2-disubstituted ethylene glycol core was started from *O*-alkylation of the diol **83** with the mesylate **82**, followed by (*R*)-epichlorohydrin. The introduction of a γ-lactone moiety was achieved by an epoxide opening reaction with the acetylide of **86**. Elimination of the secondary hydroxyl group, followed by selective hydrogenation of the resulting enyne moiety and cleavage of the MOM group, yielded the target analogue **70**. The bis-amide analogue **76** was synthesized via sequential coupling of the two carboxylic acids (**89** and **92**) with the diamine fragment **90**. The inhibitory activities against human breast cancer cell lines, MDA-MB-435 and MDA-MB-468, and non-cancerous human mammary epithelial cells (HMEC) were examined (Table 6). All analogues, with the exception of **80** and **81**, showed low micromolar potencies against MDA-MB-468, whereas they were less active against MDA-MB-435 and displayed satisfactory selectivity for the non-cancerous cell line HMEC. For example, the *N*,*N*’-dimethyl bis amide derivative **77** (SI = 69), the most potent analogue in this report, showed better selectivity for the inhibition of MDA-MB-468 and HMEC than did its parent **22c** (SI = 14). Moreover, compound **77** exhibited 30 times more potency against MDA-MB-468 cell lines than did **22c**. These results indicate that the introduction of conformational constraint was useful for the optimization of this class of anticancer agents.

**Table 5:** Summary of the inhibitory potencies (IC_50_) of the test compounds.^a^

Compounds	IC_50_ (nM)
**56**	0.83
**57**	1.9
**58**	1.0
**59**	1.4
**60**	0.90
bullatacin	0.85

^a^ The IC_50_ value is the molar concentration needed to reduce the control NADH oxidase activity (0.60–0.65 mmol NADH / min / mg of protein) in submitochondrial particles by half.

**Scheme 7:**
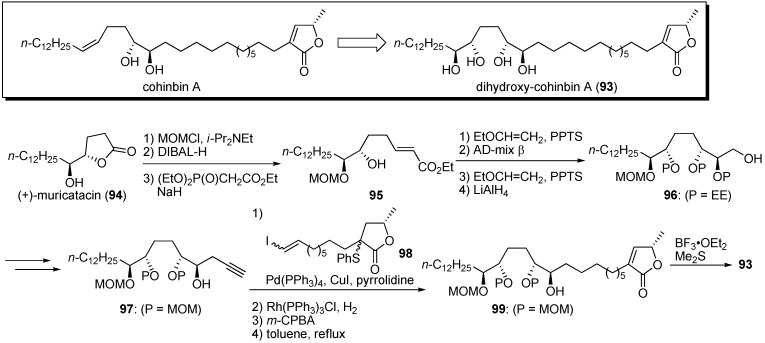
Synthesis of dihydroxy-cohinbin A by Konno and Miyoshi’s group.

Konno and Miyoshi noted that the THF ring acted as a hydrophilic anchor in the mitochondrial membrane. Dihydroxy-cohinbin A **93** was designed to increase the hydrophilicity of cohinbin A, which belongs to a class of non-THF acetogenins (Scheme 7) [36]. The synthesis of dihydroxy-cohinbin A **93** began with (+)-muricatacin **94**. The construction of the tetraol moiety was achieved by asymmetric dihydroxylation of the α,β-*E*-unsaturated ester with an AD-mix β. Tetraol fragment **97** and γ-lactone fragment **98** were connected by the Sonogashira coupling reaction. After reduction of the enyne moiety, the construction of the α,β-unsaturated-γ-lactone moiety, followed by deprotection of the MOM group, yielded dihydroxy-cohinbin A **93**. The inhibitory activities of dihydroxy-cohinbin A **93** and the intermediate **99** against bovine heart mitochondrial complex I were examined (Table 7). The intermediate **99**, which has one free and three MOM-protected hydroxyl groups, lost inhibitory activity. Dihydroxy-cohinbin A **93** showed potent inhibition at the nanomolar level, although its activity was weaker than that of bullatacin. Konno *et al*. surmised that bioactivity of dihydro-cohinbin A **93** was diminished by the high degree of flexibility in the tetraol unit compared with the THF unit bearing flanking hydroxyl groups.

**Table 6:** Bioactivity screening of newly synthesized analogues.^a^

Compounds	IC_50_[μM]		
	MDA-MB-435^b^	MDA-MB-468^c^	HMEC^d^
**22c^e^**	> 100	5.932	82.11
**70**	6.467	0.830	14.25
**71**	4.170	1.005	10.86
**72**	5.500	0.994	13.60
**73**	11.24	1.630	17.68
**74**	25.69	2.559	20.63
**75**	18.40	3.007	17.95
**76**	> 100	2.953	> 100
**77**	> 100	0.218	15.11
**78**	> 100	11.81	> 100
**79**	> 100	61.59	> 100
**80**	> 100	2.019	> 100
**81**	12.61	0.858	70.00

^a^ Inhibition of cell growth by the listed compounds for MDA-MB-435, MDA-MB-468, and HMEC cells was determined by WST (2-(2-methoxy-4-nitrophenyl)-3-(4-nitrophenyl)-5-(2,4-disulfo-phenyl)-2H-tetrazolium monosodium salt) assay; ^b^MDA-MB-435: Human breast cancer cell; ^c^ MDA-MB-468: Human breast cancer cell; ^d^ HMEC: Non-cancerous human mammary epithelial cells; ^e^ **22c** was used as a positive control.

**Table 7:** Summary of the inhibitory potencies (IC_50_) of the test compounds.

Compounds	IC_50_ (nM)
**93**	20
**99**	4100
bullatacin	0.8

## 3. Modification of the Hydrocarbon Chain

**Scheme 8:**
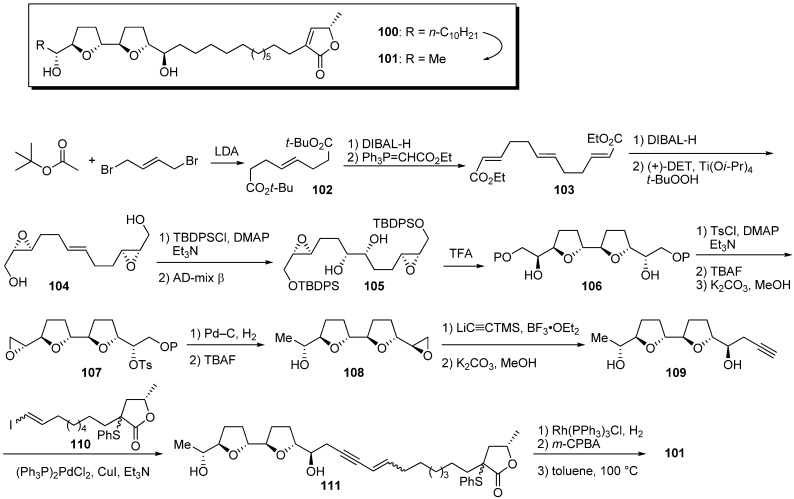
Synthesis of analogues possessing a methyl group on the left side of the THF ring by Miyoshi’s group.

Miyoshi *et al*. investigated the role of the hydrophobic alkyl chain that is a feature of natural acetogenins. First, they designed the analogue **101** possessing a methyl group on the left side of the THF ring in place of a long hydrocarbon chain (Scheme 8) [37]. The synthesis of the bis-THF fragment began with condensation of *tert*-butyl acetate and *trans*-1,4-dibromo-2-butene, giving the diester **102**. Chain extension of **102**, followed by Sharpless asymmetric epoxidation, gave the epoxy alcohol **104**. After silylation of the primary alcohol, Sharpless asymmetric dihydroxylation followed by treatment with TFA afforded the bis-THF core **106**. The bis-THF core **106** was converted to the epoxide **107** in the following sequential reactions: (1) tosylation of secondary alcohols; (2) mono-desilylation of TBDPS group; (3) treatment with K_2_CO_3_. Opening of epoxide **107** by hydrogenation with Pd–C, followed by treatment with excess TBAF, yielded alcohol **108**. After opening epoxide **108** with trimethylsilylacetylide, followed by desilylation, Pd(0)-mediated coupling of alkyne **109** with vinyl iodide **110** afforded the enyne **111**. Hydrogenation of **111** and sequential thermal elimination of the sulfide moiety gave the target analogue **101**. Although the IC_50_ of **101** (3.1 nM) against bovine heart mitochondrial complex I was weaker than the inhibitory activity of the model compound **100** (0.9 nM), the analogue **101** retained sufficiently potent inhibitory activity (Table 8). These results indicate that the large hydrophobicity of the left side of the THF ring was not essential for the activity.

**Table 8:** Summary of the inhibitory potencies (IC_50_) of the test compounds.

Compounds	IC_50_ (nM)
**100**	0.9
**101**	3.1

**Scheme 9:**
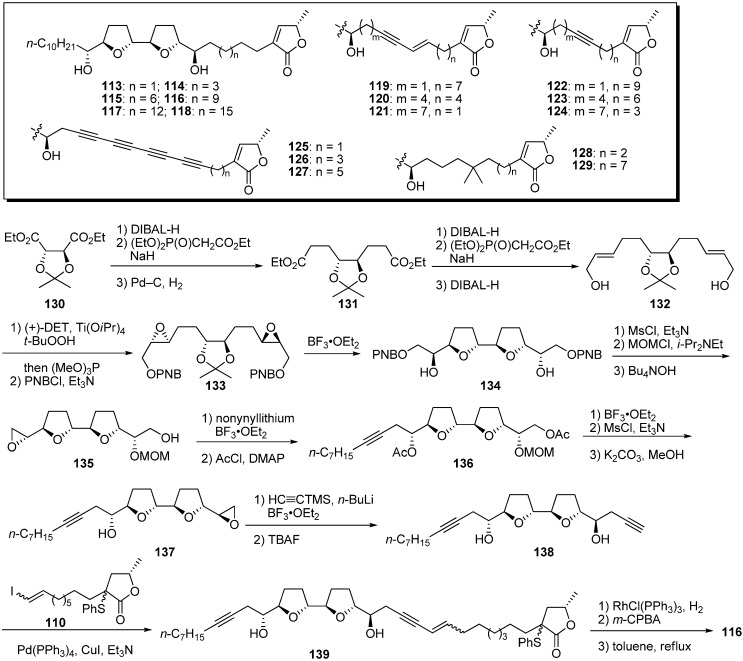
Synthesis of analogues possessing modified alkyl spacers linking the THF and γ-lactone rings by Miyoshi’s group.

Miyoshi’s group also reported modification of the alkyl spacer linking the THF and γ-lactone rings [38,39,40]. They designed a series of derivatives in which the spacer’s length was varied while other structural factors remained the same, or in which the local flexibility of the spacer was specifically reduced by introducing multiple bond(s) into different regions of the spacer. A representative synthetic pathway is given by the preparation of **116** (Scheme 9). The synthesis of the THF core **134** started from the diethyl 2,3-*O*-isopropylidene-D-tartrate **130** according to Sasaki *et al.* [41]. After sequential chain extension of **130**, Sharpless asymmetric epoxidation of the resulting allyl alcohol **132**, followed by treatment with BF_3_•OEt_2_, gave the bis-THF core **134**. Monomesylation of the secondary alcohol of **134**, followed by protection of the remaining alcohol and deprotection of the PNB group, yielded the epoxide **135**. Treatment of **135** with nonynyllithium, followed by trimethylsilylacetylide, afforded the diyne **138** via **136** and **137**. Connection of the two fragments **138** and **110** was achieved via Sonogashira coupling to give **139**. Reduction of **139**, followed by the formation of the α,β-unsaturated-γ-lactone moiety, gave the target analogue **116**. The inhibitory potency of the synthetic analogues was examined (Table 9). The optimal length of the spacer for inhibition was approximately 13 carbon atoms, which corresponds to the length of spacers in most active natural acetogenins, such as bullatacin. Elongating the spacer beyond 13 carbons reduced inhibitory activity more drastically than did shortening the spacer. Inhibitory potency was not influenced by enhancement of the hydrophobicity (**128** and **129**) or local flexibility of the spacer (**119**–**124**). Surprisingly, tetrayne analogues **125**–**127** still exhibited potent inhibition at nanomolar levels, but the double inhibitor titration of complex I activity suggested that the action site of **126** was not identical to that of common acetogenins.

**Table 9:** Summary of the inhibitory potencies (IC_50_) of the test compounds.

Compounds	IC_50_ (nM)	Compounds	IC_50_ (nM)
**113**	14	**122**	1.0
**114**	1.6	**123**	0.83
**115**	1.2	**124**	0.85
**116**	0.85	**125**	6.2
**117**	13	**126**	1.7
**118**	271	**127**	3.0
**119**	0.92	**128**	1.3
**120**	1.2	**129**	1.2
**121**	1.1		

**Figure 2:**
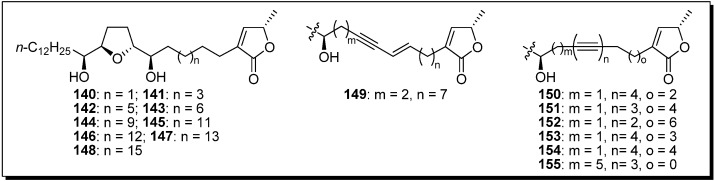
Design of analogues possessing modified alkyl spacers linking the THF and γ-lactone rings, by Miyoshi’s group.

The inhibitory potency of the mono-THF analogue (**144** vs. **150**) was drastically reduced, compared with the corresponding bis-THF analogues (**116** vs. **126**), by the introduction of a tetrayne structure into the spacer. To clarify the effect of the introduction of tetrayne, Miyoshi *et al*. synthesized a new series of tetrayne analogues **150**–**155** (Figure 2). The inhibitory activity of this series showed that the flexibility of the spacer region close to the THF ring was more important than the flexibility of the spacer near the γ-lactone (Table 10).

**Table 10:** Summary of the inhibitory potencies (IC_50_) of the test compounds.

Compounds	IC_50_ (nM)	Compounds	IC_50_ (nM)
**140**	131	**148**	1050
**141**	11	**149**	5.2
**142**	10	**150**	280
**143**	10	**151**	72
**144**	2.3	**152**	12
**145**	16	**153**	142
**146**	34	**154**	185
**147**	117	**155**	16

**Scheme 10:**
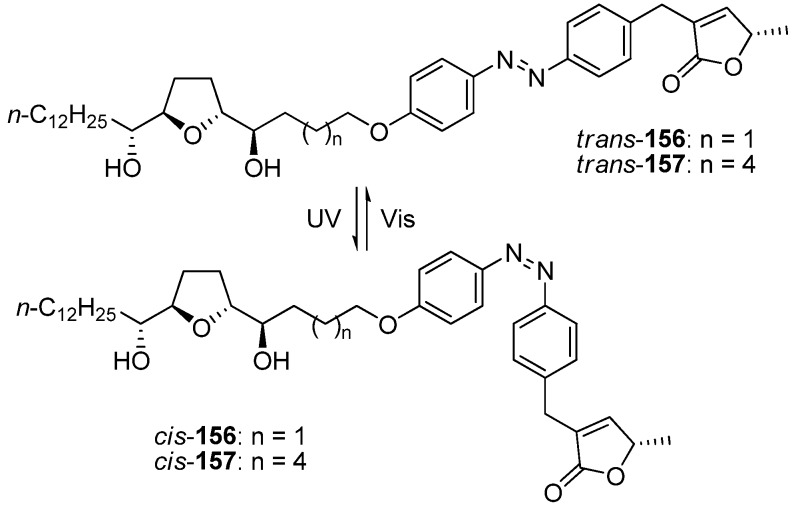
Photoresponsive analogues possessing an azobenzene moiety in the center of the spacer as a photoresponsive switch.

To gain further insight into the function of the spacer, Miyoshi *et al.* designed photoresponsive analogues **156**–**157** that had an azobenzene moiety in the center of the spacer as a photoresponsive switch (Scheme 10). The azobenzene unit of **156** reversibly *trans*–*cis* isomerized by alternating UV-visible irradiation. The NADH oxidase activity of *trans*-**156** was weaker than that of *cis*-**156**.Interestingly, the relative inhibitory effects of the *trans* and *cis*-**157**, which had a longer distance between the THF moiety and the γ-lactone moiety than did **156**, were reversed compared with those of **156**. As a result of this research, Miyoshi *et al*. suggested that acetogenins exhibited potent inhibition of complex I only when the THF moiety and the γ-lactone moiety cooperatively bound to the two putative binding sites. One of the two THF rings in bis-THF acetogenins may have served as a pseudospacer to overcome the significant structural disadvantages that arose from the spacer, whereas mono-THF acetogenins could not efficiently adapt to such structural changes.

## 4. Modification of the γ-Lactone Moiety

**Scheme 11:**
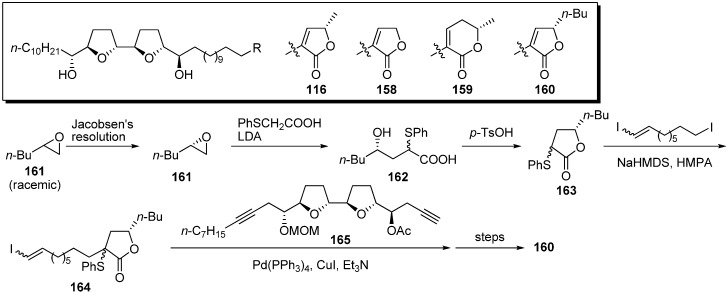
Synthesis of analogues possessing various lactones by Miyoshi’s group.

The γ-lactone moiety in acetogenins was suggested to directly interact with the target site in complex I [42]. To elucidate the role of the γ-lactone moiety, Miyoshi *et al*. synthesized analogues possessing various lactone moieties in place of the α,β-unsaturated-γ-methyl-γ-lactone in natural acetogenins (Scheme 11) [43]. A representative synthetic pathway is given by the preparation of **160**. Jacobsen’s hydrolytic kinetic resolution of the racemic epoxide **161** gave the chiral epoxide **161**. After the epoxide opening of **161** with the dianion prepared from phenylthioacetic acid, lactonization of the resulting seco acid afforded γ-butyl-γ-lactone **163**. Sequential assembly of **163**, 1,9-diiodononene, and the THF fragment **165**, followed by the usual transformation, gave the target analogue **160**. The inhibitory activities of synthetic analogues against complex I were examined (Table 11). The synthetic analogues **158**–**160** exhibited inhibition that was as potent as that of the parent compound **116**, indicating that the inhibitor binding domain in complex I may be the large cavity-like structure.

**Table 11:** Inhibition of mitochondrial complex I.

Compounds	IC_50_ (nM)
bullatacin	1.2
**116**	1.3
**158**	1.2
**159**	1.3
**160**	7.5

**Scheme 12:**
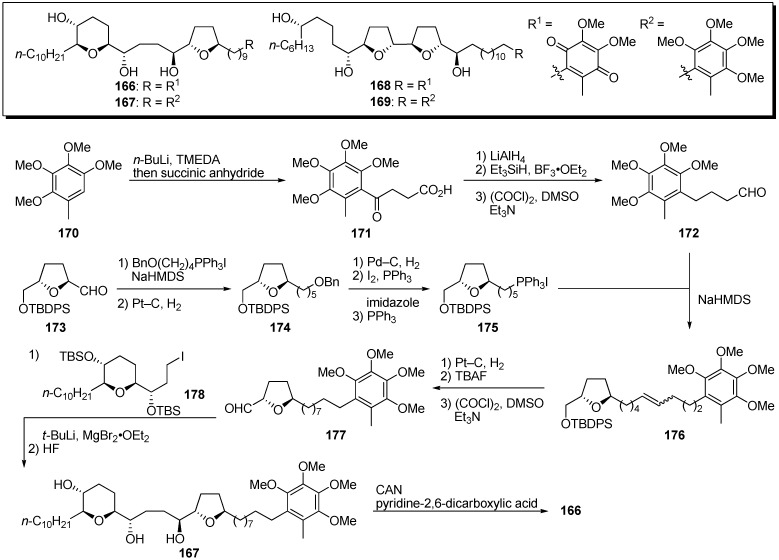
Synthesis of quinone analogues by Koert’s group.

Acetogenins were proposed to inhibit the terminal electron transfer step of mitochondrial complex I between the Fe-S cluster N2 and the ubiquinone pool [44,45,46]. The γ-lactone moiety may bind at the quinone binding site of complex I. To clarify the mode of action of acetogenins, Koert *et al*. designed quinone-mucocin **166** and quinone-squamocin D **168** in which the γ-lactone moiety was exchanged for the quinone portion of ubiquinone, the natural substrate of complex I (Scheme 12) [47,48]. A representative synthetic pathway is given by the preparation of **166**. The *ortho*-lithiation of 2,3,4,5-tetramethoxytoluene **170**, followed by treatment with succinic anhydride, gave the carboxylic acid **171**. After reduction with LiAlH_4_, deoxygenation of the resulting benzylic alcohol, followed by Swern oxidation, afforded aldehyde **172**. The THF fragment **175** was prepared from the known aldehyde **173**. After chain extension of **173** by the Wittig reaction, followed by hydrogenation, the oxygenated moiety was converted into the phosphonium salt to give **175**. Wittig reaction of the aldehyde **172** with the phosphonium salt **175** yielded alkene **176**. Introduction of the THP fragment was accomplished by stereoselective coupling of the iodide **178** with the aldehyde **177**, prepared from **176**. Deprotection of the TBS ethers gave the hydroquinone dimethyl ether **167**, which was transformed into the target quinone-mucocin **166** by oxidation with CAN. The hybrid analogues (**166**, **168**–**169**), with the exception of hydroquinone-mucocin **167**, were good inhibitors of complex I, and, in particular, quinone-mucocin **166** showed 10 times more potent activity than did mucocin (Table 12). This result indicated that the γ-lactone moiety in natural acetogenins could be exchanged for the quinone of ubiquinone, although it is unclear that the quinone moiety of the hybrid molecule accepted electrons from complex I.

**Table 12:** Inhibition of mitochondrial complex I.

Compounds	IC_50_ (nM)
mucocin	45
**166**	4.9
**167**	163
squamocin D	8.7
**168**	2.3
**169**	6.2
rotenone	1.3

**Scheme 13:**
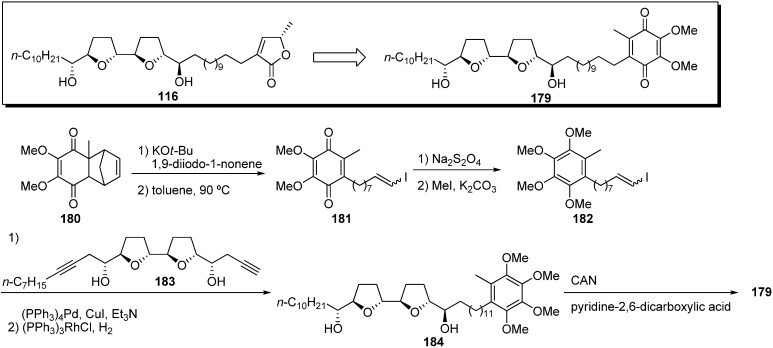
Synthesis of quinone analogues by Miyoshi’s group.

Miyoshi *et al.* also synthesized a quinone analogue **179** using the most potent acetogenin **116** synthesized in their laboratory as the mother compound (Scheme 13) [49]. Analogue **116** had inhibitory potency equal to bullatacin, the most potent acetogenin. The key fragment **182** was prepared from the known quinone **180** [50] by alkylation with 1,9-diiodo-1-nonene and a retro-Diels-Alder reaction followed by methylation of the reduced form of quinone **181**. Sonogashira coupling of the THF fragment **183** with the vinyl iodide **182**, followed by hydrogenation, gave tetramethoxytoluene **184**, which was transformed into the quinone analogue **179** by oxidation. The inhibitory activity of **179** was comparable with that of the mother compound **116** or bullatacin (Table 13). Miyoshi *et al*. suggested that the presence of a conjugated carbonyl group may be important for the inhibitory activity of complex I due to the low potency of hydroquinone-dimethyl ether **184**. Moreover, the ^13^C-labeled quinone acetogenins were also synthesized to examine the binding behavior of the quinone group with complex I [51].

**Table 13:** Inhibitory potencies of test compounds.

Compounds	IC_50_ (nM)
bullatacin	0.9
**116**	0.9
**179**	1.2
**184**	280

**Scheme 14:**
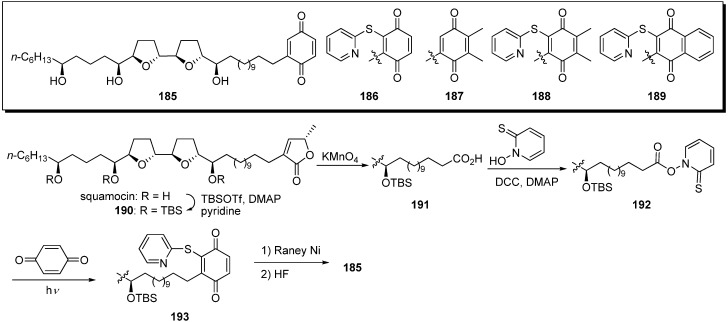
Semisynthesis of quinone analogues by Poupon’s group.

Poupon and Susin *et al.* reported the semisynthesis of quinone analogues **185**–**189** from natural squamocin (Scheme 14) [52]. A representative synthetic pathway is given by the preparation of **185**. Treatment of TBS-protected squamocin **190** with KMnO_4_ gave the carboxylic acid **191**. The condensation of **191** and thiopyridine-*N*-oxide with DCC, followed by exposure to light in the presence of the benzoquinone, afforded the thiopyridylquinone derivative **193**. After reductive desulfurization of **193** with Raney Ni, the deprotection of the tris-TBS ether yielded the target analogue **185**. Screening demonstrated that analogues **185** and **187** possessed a higher pro-apoptotic potential than natural squamocin, whereas the other analogues, **186**, **188**, and **189**, were less effective than squamocin (Table 14). Moreover, quinone analogues **185** and **187** were potent inhibitors of complex I, although the inhibition activity was weaker than that of squamocin. 

**Table 14:** Inhibition of complex I.

Compounds	IC_50_ (nM)
squamocin	1.3
rotenone	30
**185**	15
**186**	10
**190**	> 3,000

**Scheme 15:**
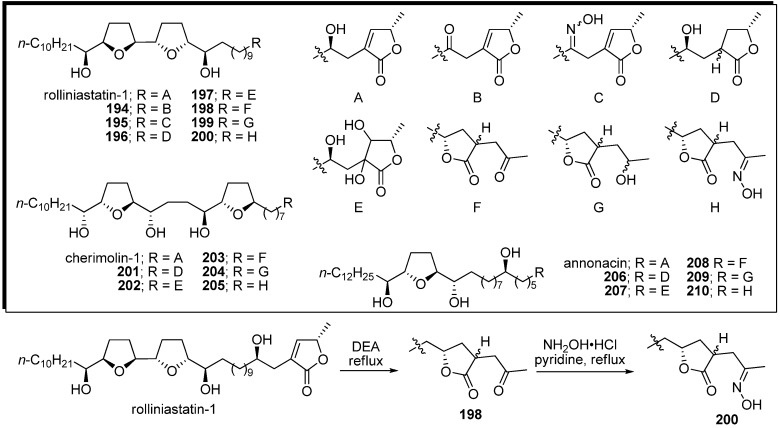
Semisynthesis of analogues modified at the α,β-unsaturated γ-methyl-γ-lactone moiety by Cortes’ group.

Cortes *et al*. reported a series of semisynthetic analogues, modified at the α,β-unsaturated γ-methyl-γ-lactone moiety (Scheme 15) [53,54,55,56]. A representative synthetic pathway is given by the preparation of **200**. Translactonization of rolliniastatin-1 by alkaline treatment [57,58] gave the isoacetogenin analogue **198** as a mixture of 2,4-*cis* and 2,4-*trans* diastereomers. The carbonyl group of **198** was transformed into the oxime **200** with NH_2_OH•HCl in pyridine. The bis-THF acetogenin analogues, with the exception of **202** and **204**, indicated more potent inhibitory activity against complex I than the natural compound (Table 15). Annonacin analogues **206**–**210** were tested against some tumor cell lines. Interestingly, the tetrahydroxyl analogue **207**, whose inhibitory potency of complex I was the weakest among the annonacin analogues, was the most potent in the cytotoxicity assays.

**Scheme 16:**
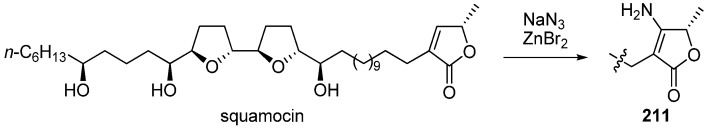
Synthesis of β-aminosquamocin by Poupon’s group.

Poupon and Brandt *et al*. reported the synthesis and biological evaluation of β-aminosquamocin **211** (Scheme 16) [59]. One-step transformation into **211** from squamocin was achieved by treatment with sodium azide and zinc bromide in boiling water. β-Aminosquamocin **211** exhibited more potent cytotoxity against KB3-1 cell lines than did squamocin, despite an inhibitory activity of **211** against complex I that was four times weaker than that of the natural compound (Table 16). Surprisingly, β-aminosquamocin **211** demonstrated inhibitory activity against complex III at nanomolar levels.

**Table 15:** Inhibitory potency against complex I.

Compounds	IC_50_ (nM)	Compounds	IC_50_ (nM)
rolliniastatin-1	0.60	**202**	8.47
**194**	0.42	**203**	0.83
**195**	0.25	**204**	2.48
**196**	0.43	**205**	0.54
**197**	0.21	annonacin	2.3
**198**	0.33	**206**	3.6
**199**	0.18	**207**	21.8
**200**	0.23	**208**	3.3
cherimolin-1	1.84	**209**	5.8
**201**	1.22	**210**	1.9

**Table 16:** Inhibitory activities of **211** and squamocin.

Compounds	Cytotoxity (KB3-1)^a^ (M)	Complex I inhibition^b^ (nM)	Complex III inhibition^c^ (nM)
squamocin	1.6 × 10^−13^	2	inactive
**211**	< 10^−14^	8	40

^a^ IC_50_ for human nasopharyngeal epithelioid carcinoma cells; ^b^ IC_50_ for NADH: *n*-Decylubiquinone oxidoreductase (bovine submitochondrial particles); ^c^ IC_50_ for *n*-decylubiquinol: Cytochrome c oxidoreductase (liposomal reconstitution of bovine enzyme).

**Scheme 17:**
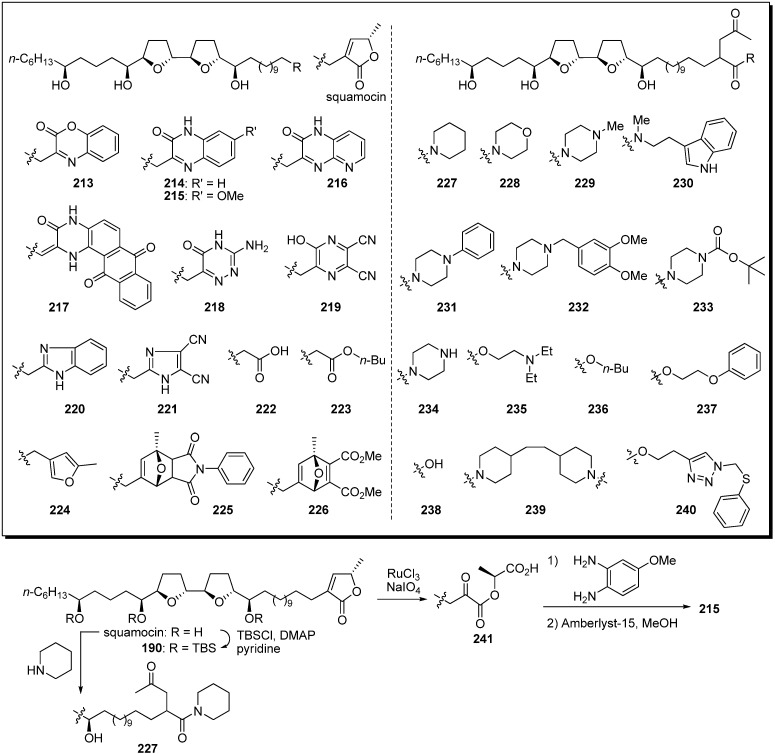
Semisynthesis of squamocin analogues by Lewin’s group.

A library of heterocyclic analogues of squamocin was semisynthesized by Lewin *et al.*, as heterocycles are commonly found as base-structures of potent complex I inhibitors (Scheme 17) [60,61,62]. A representative synthetic pathway is given by the preparation of **215** and **227**. Ruthenium-catalyzed oxidative degradation of terminal γ-lactone in tris-TBS protected squamocin **190** yielded the α-ketoester **241**. The condensation of **241** with 4-methoxy-*o*-phenylenediamine, followed by deprotection of the TBS ether, afforded the target analogue **215**. The γ-ketoamide analogue **227** was synthesized from natural squamocin by treatment with piperidine. The inhibitory activities of heterocyclic analogues **213**–**221** against complex I indicated that the γ-lactone moiety in the natural acetogenins could be exchanged for heterocycles (Table 17). In particular, the benzimidazole analogue **220** had potent inhibitory activity equal to that of squamocin. The α-ketoamide and α-ketoester derivatives, with the exception of **237**, showed potent inhibitory activity against complex I at the nanomolar level. Moreover, they had significant cytotoxic activity against KB 3-1 cell lines, although their activities were weaker than that of squamocin.

**Table 17:** Biological activities of squamocin analogues.

	**Complex I Inhibition IC_50_ (nM)**		**Cytotoxicity** (KB 3-1) IC_50_ (M)		**Complex I Inhibition IC_50_** **(nM)**		**Cytotoxicity** (KB 3-1) IC_50_ (M)
	NADH oxidase	NADH:DB oxidoreductase			NADH oxidase	NADH:DB oxidoreductase	
squamocin	0.8–0.9	1.3	1.8 × 10^−13^	**227**	41	nt	4.0 × 10^−7^
**213**	nt	8.1	nt	**228**	22	nt	3.6 × 10^−7^
**214**	2.0	7.9	nt	**229**	nt	nt	4.0 × 10^−7^
**215**	nt	10	nt	**230**	52	nt	3.9 × 10^−7^
**216**	nt	17	nt	**231**	nt	nt	1.5 × 10^−8^
**217**	13	nt	nt	**232**	74	154	1.2 × 10^−8^
**218**	19	nt	nt	**233**	nt	nt	4.1 × 10^−8^
**219**	122	nt	nt	**234**	nt	nt	5 × 10^−7^
**220**	0.9	3.3	nt	**235**	13	nt	7.5 × 10^−10^
**221**	14	41	nt	**236**	17	nt	4.6 × 10^−10^
**222**	6.2	38	2.8 × 10^−7^	**237**	> 3000	nt	8.1 × 10^−8^
**223**	2.3	9.2	6.8 × 10^−8^	**238**	12	nt	1.3 × 10^−9^
**224**	2.4	nt	3.2 × 10^−8^	**239**	26	nt	2.7 × 10^−8^
**225**	30	nt	3.5 × 10^−7^	**240**	12	nt	5.1 × 10^−9^
**226**	23	nt	3.0 × 10^−8^				

**Scheme 18:**
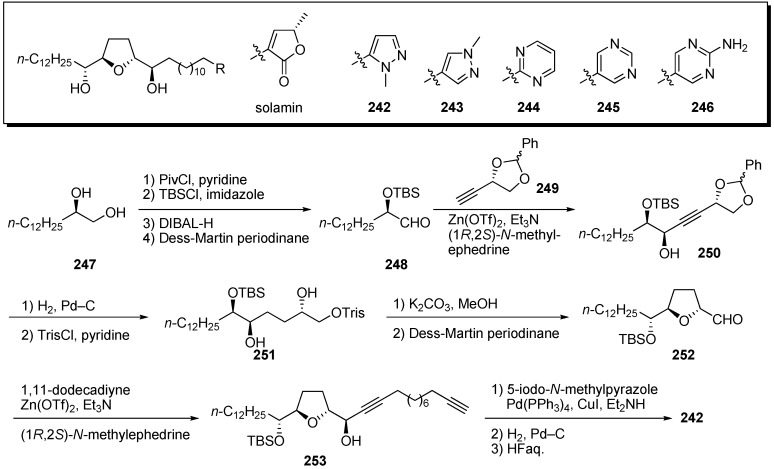
Synthesis of heterocyclic solamin analogues by Tanaka’s group.

Kojima and Tanaka *et al*. designed and synthesized heterocyclic analogues of solamin, a simple mono-THF acetogenin (Scheme 18) [63]. A representative synthetic pathway is given by the preparation of **242**. 

1,2-Diol **247** was converted to the α-TBS-oxyaldehyde **248** via the following sequential reactions: (1) Esterification of the primary alcohol; (2) silylation of the remaining secondary alcohol; (3) deprotection of pivaloyl ester; 4) oxidation of the resulting primary alcohol. The introduction of a chiral C4-unit **249** was achieved by asymmetric alkynylation with a Zn(OTf)_2_/Et_3_N system to give **250** [64]. Reduction of the triple bond and deprotection of the benzylideneacetal, followed by selective sulfonylation of the primary alcohol, gave sulfonate **251**. Treatment of **251** with K_2_CO_3_, followed by oxidation of the resulting primary alcohol, afforded the THF ring **252**. Alkynylation of **252** with 1,11-dodecadiyne proceeded smoothly to give the propargy alcohol **253**. After introduction of the aromatic ring by the Sonogashira reaction, hydrogenation of diyne moiety, followed by deprotection, yielded the target analogue **242**. Synthetic analogues **242**–**246** were tested for *in vitro* antiproliferative activity against a panel of 39 human cancer cell lines [65]. Selected GI_50_ (concentration for 50% inhibition of cell growth relative to control) values are summarized in Table 18. The *N*-methylpyrazole derivative **242** displayed strong cytotoxicity against NCI-H23 with potencies that were 80 times higher than those of solamin. These results indicate that the γ-lactone moiety could be exchanged for a heterocycle and that antitumor agents more effective than the natural acetogenins could be produced.

**Table 18:** Selected GI_50_ (M) values of heterocyclic analogues against human cancer cell lines.

Compounds	MCF-7^a^	SF-295^b^	HCT-116^c^	NCI-H23^d^	OVCAR-4^e^	MKN7^f^	PC-3^g^
solamin	> 10^−4^	4.0 × 10^−5^	> 10^−4^	7.3 × 10^−5^	> 10^−4^	1.3 × 10^−5^	> 10^−4^
**242**	2.7 × 10^−5^	2.4 × 10^−5^	9.2 × 10^−6^	9.1 × 10^−7^	3.7 × 10^−5^	6.2 × 10^−6^	> 10^−4^
**243**	> 10^−4^	2.4 × 10^−5^	7.0 × 10^−5^	1.3 × 10^−5^	> 10^−4^	5.4 × 10^−6^	> 10^−4^
**244**	7.9 × 10^−5^	> 10^−4^	> 10^−4^	2.8 × 10^−5^	3.8 × 10^−5^	2.7 × 10^−5^	6.8 × 10^−5^
**245**	3.4 × 10^−5^	2.5 × 10^−5^	2.8 × 10^−5^	1.1 × 10^−5^	2.2 × 10^−5^	6.1 × 10^−6^	3.5 × 10^−5^
**246**	> 10^−4^	5.6 × 10^−5^	> 10^−4^	3.6 × 10^−5^	> 10^−4^	9.4 × 10^−6^	> 10^−4^

^a^ breast cancer; ^b^ central nervous system cancer; ^c^ colon cancer; ^d^ lung cancer; ^e^ ovarian cancer; ^f^ stomach cancer; ^g^ prostate cancer.

**Scheme 19:**
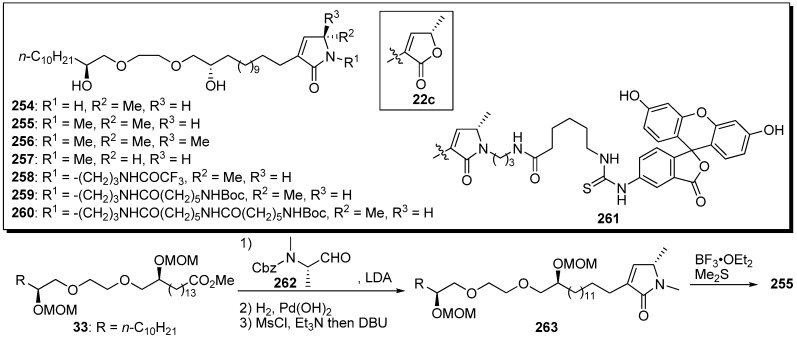
Synthesis of lactam-containing analogues by Yao’s group.

Yao *et al.* reported the synthesis of new analogues bearing a terminal lactam moiety instead of the γ-lactone in their original polyether mimics **22c** (Scheme 19) [66]. A representative synthetic pathway is given by the preparation of **255**. Aldol reaction of the enolate from the ester **33** with amino aldehyde **262**, removal of the Cbz group, and *in situ* cyclization with β-elimination gave the lactam **263**. Deprotection of the MOM ethers afforded the target compound **255**. *N*-Methyl substituted lactam derivatives **255**–**257** showed potent cytotoxicity and good selectivity between human cells and tumor cells (Table 19). Unfortunately, analogues **258**–**260**, with different length of the linkers at the nitrogen atom of the terminal lactam, showed dramatically decreased cytotoxic activity compared with **255**, and no activity was measured in the fluorescent probe **261**.

**Table 19:** Cytotoxicity screening of lactam-containing analogues.

Compounds	IC_50_ (µM)			
	Chang	B16	BEL-7404	SK-Hepl
**22c**	NA^a^	0.035	0.041	0.065
**254**	NA	0.87	2.20	NA
**255**	NA	0.013	0.234	0.589
**256**	NA	0.478	0.845	0.583
**257**	NA	0.168	0.168	0.104
**258**	NA	0.995	1.35	NA
**259**	NA	NA	3.20	NA
**260**	NA	NA	2.50	NA
**261**	NA	NA	NA	NA

^a^ not active.

**Scheme 20:**
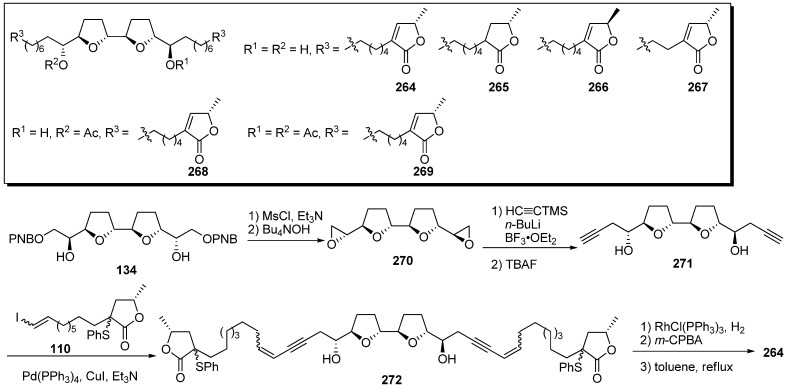
Synthesis of analogues possessing two γ-lactone moieties connected to the bis-THF ring by flexible alkyl chains.

Miyoshi *et al*. designed analogues possessing two γ-lactone moieties connected to the bis-THF ring by flexible alkyl chains with the expectation that these analogues would elicit inhibitory activity that was twice as potent as that of ordinary acetogenins [38], because the γ-lactone moiety was suspected to interact directly with the binding site of complex I. A representative synthetic pathway is given by the preparation of **264** (Scheme 20). Mesylation of the secondary alcohols of the bis-THF **134**, followed by deprotection of the PNB groups, gave bis-epoxide **270**. After epoxide opening of **270** with trimethylsilylacetylide, treatment with TBAF afforded diyne **271**. The connection of **271** with the γ-lactone fragment **110** was carried out via the Sonogashira reaction. Reduction of **272**, followed by the formation of the α,β-unsaturated-γ-lactone moiety, gave the target analogue **264**. The inhibitory activities of the synthetic analogues against complex I were measured (Table 20). The inhibitory activities of analogues **264**–**267** were identical to the activity of bullatacin. These results indicate that the analogues **264**–**269** does not work as two mole of inhibitors, although they have two mole of γ-lactone moieties in their one molecule, suggesting in turn that one γ-lactone and one THF moieties act cooperatively on the enzyme.

**Table 20:** Inhibition of complex I.

Compounds	IC_50_ (nM)	Compounds	IC_50_ (nM)
**264**	1.2	**268**	2.0
**265**	1.6	**269**	18
**266**	1.2	bullatacin	1.2
**267**	1.9		

**Scheme 21:**
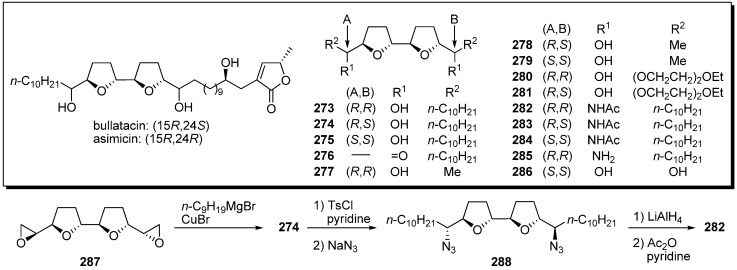
Synthesis of analogues without the γ-lactone moiety by Sasaki’s group.

Sasaki and Maeda *et al.* tested the assertion that acetogenins possessed affinity toward metal cations. Such properties may be related to their biological activity, because acetogenins are structurally analogous to known ionophores, such as oligo-tetrahydrofurans [67]. They designed analogues possessing only a bis-THF moiety without the γ-lactone moiety (Scheme 21) [25,26,68]. A representative synthetic pathway is given by the preparation of **282**. Epoxide opening of **287** with a Grignard reagent gave the diol **274**. Tosylation of two secondary alcohols followed by azidation afforded the azide **288**. After reduction of the azide groups, acetylation of the resulting secondary amines yielded the target analogue **282**. Complexation properties of natural acetogenins and analogues were investigated by ^1^H NMR titration (Table 21). Among the bis-THF analogues with flanking hydroxyl groups, it was revealed that some analogues (**274** and **277**) had selective affinity towards Ca^2+^. Very high binding affinity was exhibited by diacetamide **284** to both Mg^2+^ and Ca^2+^ in the formation of 2:1 ligand-to-metal complexes.

**Table 21:** Binding properties of the bis-THF ligands.

Compounds	Metal	Compound/metal	*K*s (10^3^ M^−1^)	Compounds	Metal	Compound/metal	*K*s (10^3^ M^−1^)
bullatacin	Ca^2+^	2:1	3.10	**280**	Ca^2+^	1:1	0.08
asimicin	Ca^2+^	4:1	5.50	**280**	Mg^2+^	1:1	0.62
**273**	Ca^2+^	4:1	0.15	**281**	Ca^2+^	1:1	0.06
**273**	Mg^2+^	4:1	0.09	**281**	Mg^2+^	1:1	0.11
**273**	K^+^	4:1	0.13	**282**	Ca^2+^	4:1	0.66
**273**	Na^+^	4:1	0.04	**282**	Mg^2+^	2:1	4.80
**274**	Ca^2+^	4:1	1.50	**282**	K^+^	4:1	0.25
**274**	Mg^2+^	4:1	0.21	**282**	Na^+^	4:1	0.28
**275**	Ca^2+^	4:1	0.10	**283**	Ca^2+^	4:1	9.60
**277**	Ca^2+^	2:1	9.00	**283**	Mg^2+^	4:1	3.00
**277**	Mg^2+^	2:1	0.04	**283**	K^+^	2:1	1.00
**277**	K^+^	–	–	**283**	Na^+^	4:1	1.20
**277**	Na^+^	–	–	**284**	Ca^2+^	2:1	> 100
**278**	Ca^2+^	2:1	0.83	**284**	Mg^2+^	2:1	> 100
**278**	Mg^2+^	2:1	0.06	**284**	K^+^	4:1	1.60
**278**	K^+^	–	–	**284**	Na^+^	4:1	3.40
**278**	Na^+^	–	–				
**279**	Ca^2+^	2:1	0.05				
**279**	Mg^2+^	2:1	0.07				
**279**	K^+^	–	–				
**279**	Na^+^	–	–				

The GI_50_ values of their analogues against cancer cell lines are listed in Table 22 [69]. Although the analogues possessing short alkyl chains **277**–**279**, ether chain **280**–**281**, or no chain, **286**, did not show activity, the analogues **273**–**275** with long alkyl chains retained cytotoxicity against P388 cells. The analogues (**282**–**283** and **285**) with amino groups instead of hydroxyl groups also showed inhibitory activity against cancer cell lines.

**Table 22:** GI_50_ (μM) values against cancer cell lines.

Compounds	P388^a^	PC-6^b^	NUGC-3^c^	Compounds	P388^a^	PC-6^b^	NUGC-3^c^
bullatacin	1.04 × 10^−4^	> 0.250	> 0.250	**278**	11.6	> 50.0	−
asimicin	3.51 × 10^−4^	> 0.250	> 0.250	**279**	> 25.0	> 25.0	> 25.0
**273**	0.271	6.34	–	**280**	> 50.0	> 50.0	> 50.0
enantio-**273**	1.42	16.4	–	**281**	35.9	> 50.0	> 50.0
**274**	0.111	34.6	–	**282**	1.82	> 2.50	> 2.50
enantio-**274**	3.10	> 25.0	–	**283**	0.460	> 2.50	> 2.50
**275**	0.140	21.9	–	**284**	> 2.50	> 2.50	> 2.50
**276**	> 5.00	–	–	**285**	0.610	0.484	0.722
**277**	> 2.50	> 25.0	> 2.50	**286**	17.2	28.0	–

^a^ mouse leukemia; ^b^ human lung cancer; ^c^ human gastric cancer.

**Figure 3:**
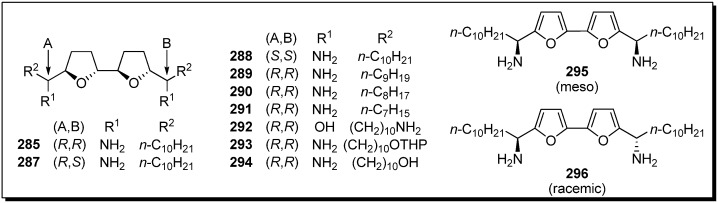
Diamino-bis-THF analogues by Sasaki’s group.

Sasaki *et al*. tested the possibility that their bis-THF analogues were active DNA binding agents, because a helix-like conformation was suggested in the studies of naturally occurring bis-THF acetogenins [70,71]. The DNA binding affinities of bis-THF analogues were evaluated by Sasaki *et al*. (Figure 3) [72]. It was revealed that the stereochemistries around the bis-THF moiety and the length of alkyl chains affected the binding affinity. The bis-furan **295** also displayed high affinity against CA12.

**Table 23:** Comparison of DNA binding affinities.^a^

**Compounds**	**C_50_ (μM)**		**Compounds**	**C_50_ (μM)**	
	CT12^b^	CA12^ c^		CT12^b^	CA12^c^
**285**	7	5.5	**292**	300	330
**287**	19	13	**293**	32	27
**288**	30	30	**294**	490	540
**289**	34	23	**295**	17	4
**290**	135	125	**296**	> 100	> 100
**291**	760	880	distamycin	19	> 15

^a^ 1.5 μM DNA and 1.5 μM ETBr were used in the buffer containing 9.4 mM NaCl, 2.0 mM HEPES, 10 mM EDTA, pH 7.0; ^b^ K_ETBr_ = 2.4 × 10^6^; ^c^ K_ETBR_ = 7.6 × 10^6^.

**Scheme 22:**
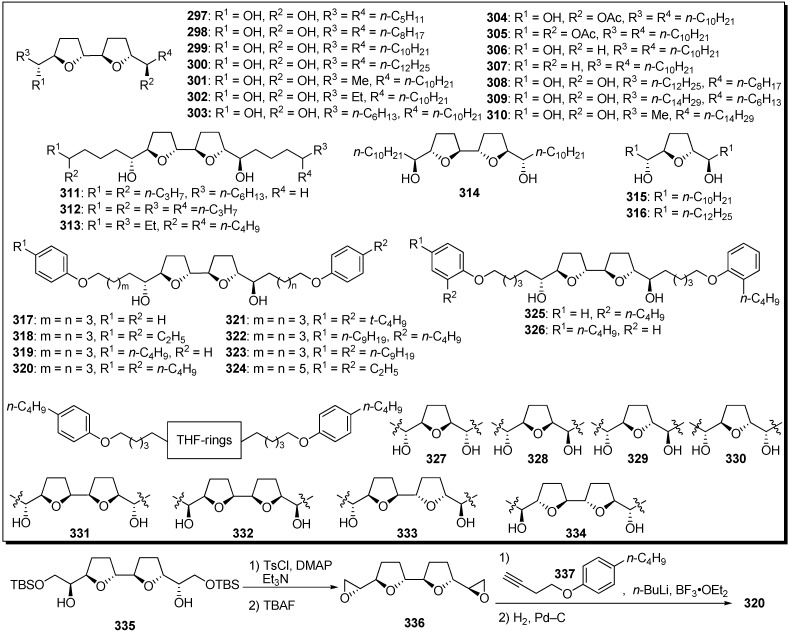
Synthesis of analogues possessing two alkyl chains without γ-lactone by Miyoshi’s group.

Surprisingly, Miyoshi *et al*. discovered that the analogues that possessed two alkyl tails without a γ-lactone in bis-THF acetogenins showed potent inhibitory activity against mitochondrial complex I (Scheme 22) [73,74,75,76,77,78,79]. A representative synthetic pathway is given by the preparation of **320**. Tosylation of secondary alcohols of bis-THF cores **335**, followed by treatment with TBAF, gave the bis-epoxide **336**. Epoxide opening of **336** with the acetylide generated from **337**, followed by hydrogenation, afforded the target analogue **320**. Inhibitory activities of synthetic analogues against complex I were examined (Table 24). The inhibitory potencies were affected by the length of the alkyl tails, and the analogue **299**, possessing unbranched decyl groups, showed the most potent activity among analogues **297**–**314**. Analogues **317**–**321**, possessing a phenol moiety at the end of the alkyl chains, exhibited more potent inhibitory activity. It was revealed that the stereochemistry around the THF ring moiety significantly influenced the inhibitory effect of the analogues. A mode-of-action study suggested that the binding site of the analogues is not identical to the binding site of ubiquinone and is downstream of the binding site of ordinary inhibitors.

**Scheme 23:**
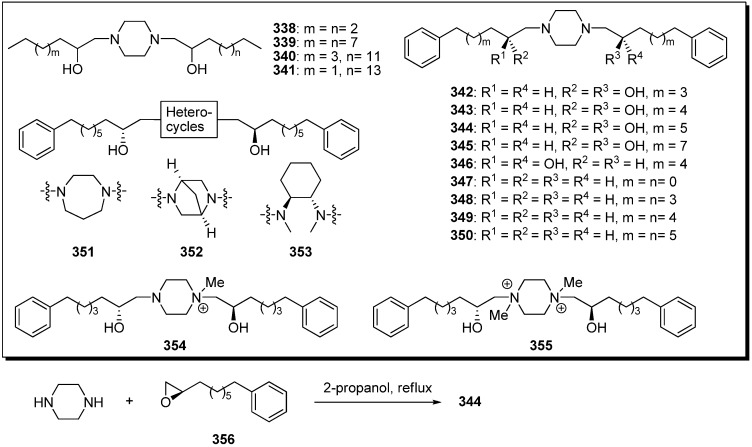
Synthesis of analogues possessing piperazines instead of the bis-THF moiety by Miyoshi’s group.

Based on the structural similarities between the hydroxylated bis-THF moiety and the piperazine ring, Miyoshi *et al*. designed a series of piperazine derivatives by replacing the bis-THF moiety of their analogues **297**–**334** with piperazine rings (Scheme 23) [80]. Piperazine analogues (e.g., **344**) were easily prepared by the condensation of piperazine and epoxide **356**. The inhibitory activities of synthetic analogues against complex I were examined (Table 25). Some piperazine analogues (**339**, **343**–**346**) showed potent inhibitory activity equal to that of the parent compounds (e.g., **320**). Although inhibitory potencies were affected by the length of alky chains as well as parent compound, the presence of two hydroxyl groups was not crucial for activity. Modifying the conformational properties of the piperazine rings did not affect activity. The photoaffinity labeling study of new piperazine derivatives revealed that this analogue bound to the 49 kDa subunit and an unidentified subunit (not ND1) with a frequency of ~1:3, but prevented the specific binding of [^125^I](trifluoromethyl)phenyldiazirinyl acetogenin to the ND1 subunit.

**Table 24:** Summary of the inhibitory potencies against complex I.

Compounds	IC_50_ (nM)	Compounds	IC_50_ (nM)	Compounds	IC_50_ (nM)	Compounds	IC_50_ (nM)
**297**	4500	**307**	620	**317**	1.4	**327**	39
**298**	45	**308**	7.5	**318**	1.1	**328**	41
**299**	1.6	**309**	34	**319**	0.91	**329**	47
**300**	9.0	**310**	410	**320**	0.83	**330**	38
**301**	280	**311**	27	**321**	1.0	**331**	16
**302**	45	**312**	1500	**322**	150	**332**	9.0
**303**	3.2	**313**	870	**323**	> 500	**333**	5.2
**304**	5.5	**314**	7.5	**324**	3.0	**334**	3.8
**305**	330	**315**	308	**325**	> 1000	bullatacin	0.85
**306**	14	**316**	> 25000	**326**	16		

**Table 25:** Summary of the inhibitory potencies against complex I.

Compounds	IC_50_ (nM)	Compounds	IC_50_ (nM)
**338**	> 22000	**347**	1300
**339**	2.6	**348**	12
**340**	26	**349**	2.2
**341**	110	**350**	3.6
**342**	12	**351**	1.7
**343**	1.7	**352**	2.8
**344**	1.2	**353**	670
**345**	5.9	**354**	1100
**346**	2.3	**355**	4700

## 5. Modification of the Oxygenated Moiety on Alkyl Chains

**Scheme 24:**
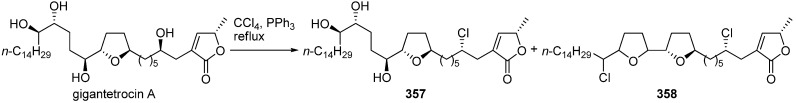
Semisynthesis of chlorinated acetogenins by McLaughlin’s group.

McLaughlin *et al.* reported a semisynthesis of chlorinated analogues, **357**–**358**, of gigantetrocin A (Scheme 24) [81]. Gigantetrocin A was refluxed with PPh_3_ in CCl_4_ to give a mixture of 4-chloro-4-deoxygigantetrocin A **357** and dichloro bis-THF derivative **358**. Both chlorinated analogues **357**–**358** showed decreased bioactivity against tumor cell lines compared with gigantetrocin A (Table 26). However, the 4-chloro derivative **357** was selectively cytotoxic to HT-29, and **358** was selectively cytotoxic to PC-3.

**Table 26:** Bioactivity data ofchlorinated analogues **357**–**358** (LC_50_ and ED_50_: μg/mL).

Compounds	BST^a^	A-549^b^	MCF-7^c^	HT-29^d^	A-498^e^	PC-3^f^	PaCa-2^g^
gigantetrocin A	2.6	2.5 × 10^−1^	6.3 × 10^−1^	4.1 × 10^−5^	nt	nt	nt
**357**	54.6	> 10	5.74	3.8 × 10^−1^	> 10	> 10	> 10
**358**	31.9	2.39	> 10	> 10	2.47	7.55 × 10^−1^	1.16
adriamycin	nt	2.43 × 10^−2^	2.09 × 10^−1^	3.46 × 10^−2^	6.49 × 10^−3^	2.62 × 10^−2^	7.90 × 10^−3^

^a^ brine shrimp lethality test; ^b^ human lung carcinoma; ^c^ human breast carcinoma; ^d^ human colon adenocarcinoma; ^e^ human kidney carcinoma; ^f^ human prostate adenocarcinoma; ^g^ human pancreatic carcinoma.

**Scheme 25:**
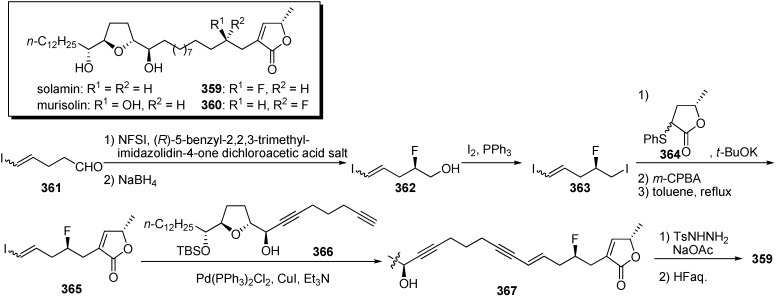
Synthesis of C4-fluorinated solamins by Tanaka’s group.

The presence of a C4-hydroxyl group in acetogenins is known to influence cytotoxic activity against tumor cell lines. Kojima and Tanaka *et al*. were interested in whether C4-fluorinated solamin showed similar activity to that of solamin or murisolin (C4-(*R*)-hydroxyl solamin), because fluorine atoms are known to mimic hydrogen atoms. Substitution of the hydroxyl group with an isoelectronic function such as fluorine in biologically active compounds can produce more potent analogues (Scheme 25) [82]. The synthesis of **359** started from enantioselective α-fluorination [83] of aldehyde **361**, followed by reduction of the aldehyde, to give the alcohol **362**. After iodination of the primary alcohol of **362**, the coupling reaction with the γ-lactone **364** and the sequential thermal elimination of the sulfide moiety afforded α,β-unsaturated γ-lactone **365**. Assembly of two fragments, **365** and **366**, prepared by a systematic construction strategy [84,85,86,87,88,89,90,91,92], with the Sonogashira reaction, followed by selective reduction of endiyne and deprotection of the TBS ether, gave the target fluorinated analogue **359**. Synthetic analogues **359**–**360** were tested for *in vitro* antiproliferative activity against a panel of 39 human cancer cell lines (Table 27). Selected results are summarized in Table 27. Two fluorinated analogues, **359** and **360**, displayed inhibitory potency levels that fell between the potencies of solamin and murisolin. Interestingly, the analogue **359** showed approximately 20 times higher cytotoxicity to MKN7 than did **360**, implying that the stereochemistry of the fluorine atom at the C4 position was recognized by cancer cell lines.

**Table 27:** Selected GI_50_ (M) values of fluorinated analogues against 39 human cancer cell lines.

Compounds	MCF-7^a^	SF-295^b^	HCT-116^c^	NCI-H23^d^	DMS114^d^	MKN7^e^	PC-3^f^
solamin	7.1 × 10^−5^	4.0 × 10^−5^	> 10^−4^	7.3 × 10^−5^	4.3 × 10^−6^	1.3 × 10^−5^	> 10^−4^
murisolin	3.9 × 10^−6^	7.3 × 10^−6^	3.7 × 10^−6^	2.2 × 10^−7^	< 10^−8^	7.0 × 10^−7^	1.7 × 10^−5^
**359**	5.6 × 10^−5^	3.7 × 10^−5^	2.0 × 10^−6^	2.3 × 10^−5^	2.6 × 10^−7^	9.6 × 10^−7^	> 10^−4^
**360**	3.7 × 10^−5^	3.1 × 10^−5^	2.7 × 10^−5^	5.1 × 10^−6^	4.6 × 10^−7^	1.9 × 10^−6^	8.4 × 10^−5^

^a^ breast cancer; ^b^ central nervous system cancer; ^c^ colon cancer; ^d^ lung cancer; ^e^ stomach cancer; ^f^ prostate cancer.

Semisynthesis of analogues possessing substituted functionalities in place of the hydroxyl groups were independently reported by Cortes’s group [53,93,94,95] and Figadère’s group [96]. 

A representative synthetic pathway is given by the preparation of **374**. Treatment of squamocin with mesyl chloride in pyridine gave the mesylate **371**. Azidation of **371**, followed by Staudinger reduction, afforded the target analogue **374**. The inhibitory potency of analogues against complex I were examined by Cortes [53,93,94,95]. The oxo and hydroxyimino derivatives showed potent activity, but the activities of acetyl, mesyl, and azide analogues were diminished. The cytotoxicities of analogues against cancer cell lines were evaluated by Figadère *et al*. [96]. All of the tested squamocin analogues exhibited much lower cytotoxicity than did natural products. On the other hand, Cortes’s group reported that guanacone analogus **381**–**386** showed more potent cytotoxicity against human cancer cell lines than parent compound [94,95].

**Scheme 26:**
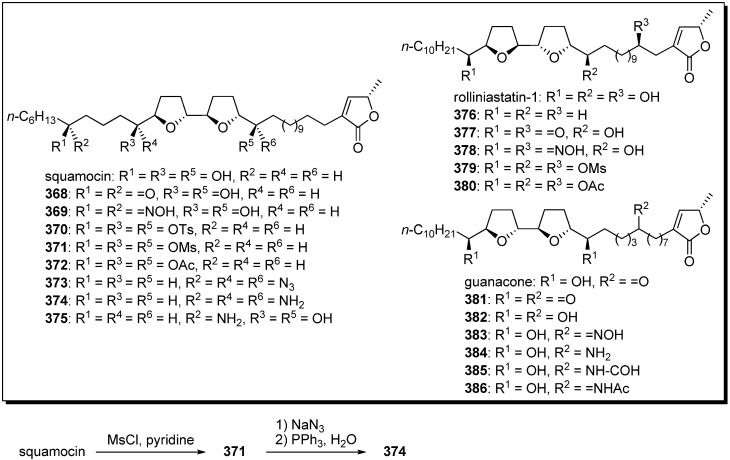
Semisynthesis of acetogenin analogues modified by an oxygenated moiety on the alkyl chains by Cortes’ group and Figadère’s group.

**Table 28:** Inhibitory potency against complex I and cytotoxicity against cancer cell lines.

**Compounds**	**IC_50_ (nM) NADH oxidase**	**EC_50_ (μM)**		**Compounds**	**IC_50_ (nM) NADH oxidase**	**EC_50_ (μM)**	
		KB	VERO			KB	VERO
squamocin	0.59	1.6 × 10^−5^	4.8 × 10^−2^	rolliniastatin-1	0.60	< 1.6 × 10^−7^	1.1 × 10^−2^
**368**	0.65	nt	nt	**376**	nt	3.5 × 10^−1^	1.4 × 10^−1^
**369**	0.74	nt	nt	**377**	0.34	nt	nt
**370**	nt	< 1.3 × 10^−1^	< 1.3 × 10^−1^	**378**	0.34	nt	nt
**371**	14.1	1.5 × 10^−2^	< 10^−1^	**379**	1.68	nt	nt
**372**	5.0	nt	nt	**380**	1.46	nt	nt
**373**	18	< 1.4 × 10^−1^	< 1.4 × 10^−1^	guanacone	1.52	nt	nt
**374**	nt	2.4× 10^−2^	< 1.6 × 10^−1^	**381**	1.65	nt	nt
**375**	nt	9.7 × 10^−2^	< 1.6 × 10^−1^	**382**	0.95	nt	nt
				**383**	0.34	nt	nt
				**384**	12.8	nt	nt
				**385**	5.5	nt	nt
				**386**	nt	nt	nt

Table 28 shows that the transformation of the secondary alcohols into acetate or mesylate led to a disappearance of cytotoxicity, but such derivatives retained strong activity against mitochondrial complex I. 

**Scheme 27:**
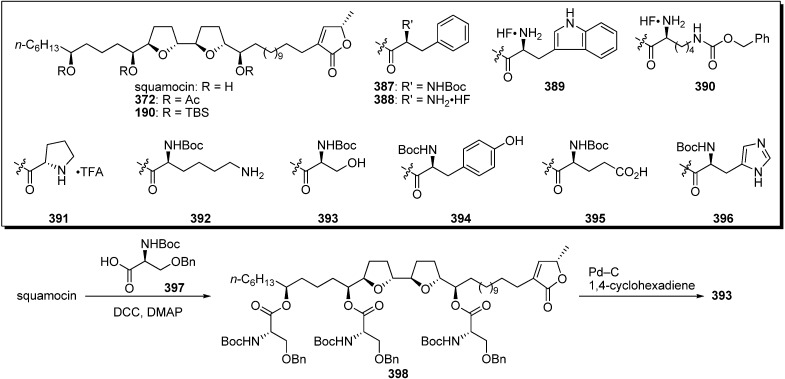
Semisynthesis of aminoacyl triester of squamocin by Lewin’s group.

To explain this ambiguity, Lewin *et al*. carried out semisynthesis and evaluation of biological activities of aminoacyl squamocin (Scheme 27) [97]. A representative synthetic pathway is given by the preparation of **393**. The esterification of squamocin with the *N*-boc-protected serine **397** gave the triester **398**. Deprotection of tri-benzyl ether using Pd–C/1,4-cyclohexadiene system afforded the target analogue **393**. The inhibitory activity against complex I and cytotoxicity against KB 3-1 cell lines were examined (Table 29). Despite enhanced polarity compared with the natural acetogenins, all derivatives showed strongly reduced activities, although their analogues were more strongly active than was the lipophilic trisilyl ether **190**.

**Table 29:** Inhibitory potency against complex I and cytotoxicity against cancer cell lines.

Compounds	IC_50_ (nM) NADH oxidase	IC_50_ (nM)	Compounds	IC_50_ (nM) NADH oxidase	IC_50_ (nM)
		KB 3-1			KB 3-1
squamocin	0.8	2.6 × 10^−14^	**391**	247.7	2.0 × 10^−8^
**372**	5.0	nt	**392**	nt	1.1 × 10^−6^
**190**	> 5000	> 10^−5^	**393**	nt	5.5 × 10^−7^
**387**	625	nt	**394**	nt	> 10^−5^
**388**	440.0	6.1 × 10^−8^	**395**	nt	> 10^−5^
**389**	nt	2.4 × 10^−7^	**396**	nt	1.7 × 10^−6^
**390**	nt	6.3 × 10^−8^			

**Scheme 28:**
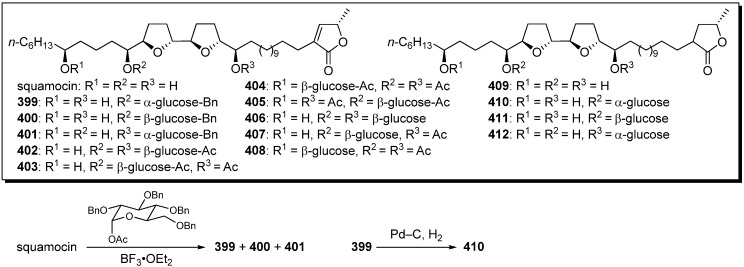
Semisynthesis of glycosyl analogues of squamocin by Figadère’s group.

To investigate the influence of aqueous solubility of acetogenins on their cytotoxicity, a series of glycosyl analogues of squamocin was synthesized by Figadère’s group (Scheme 28) [98]. A representative synthetic pathway is given by the preparation of **410**. Lewis acid catalyzed glycosylation of squamocin with 1-acetyl-2,3,4,6-tetrabenzyl-α-D-glucopyranose gave a mixture of **399**-**401**. After purification by HPLC, hydrogenation of the compound **399** gave target analogue **410**. Cytotoxicity against KB, VERO, and L1210 cell lines were examined (Table 30). Glycosylated analogues showed low activity against the normal cell (VERO) while still active against cancer cell lines (KB and L1210). The water solubility diminished the cytotoxicity probably because of low lipophilicity to cross the cell membrane. Interestingly, two analogues **401** and **411** have shown significant inhibition of the proliferation of L1210 in the G1 phase, whereas squamocin was not specific.

**Table 30:** Cytotoxic activity of glycosyl analogues of squamocin by Figadère’s group.

Compounds	EC_50_ (μM)			Compounds	EC_50_ (μM)		
	KB^a^	VERO^b^	L1210^c^		KB^a^	VERO^b^	L1210^c^
squamocin	1.6 × 10^−5^	1.6 × 10^−2^	< 4.0 × 10^−4^	**408**	1.1 × 10^−2^	3.4 × 10^−1^	1.7 × 10^−2^
**399**	4.3 × 10^−1^	8.7 × 10^−1^	nt	**409**	2.4 × 10^−4^	1.6 × 10^−2^	< 2.5 × 10^−4^
**400**	nt	nt	5.0 × 10^−2^	**410**	1.9 × 10^−3^	6.0 × 10^−1^	1.0 × 10^−2^
**404**	9.6 × 10^−4^	2.0 × 10^−2^	3.0 × 10^−1^	**411**	nt	nt	8.0 × 10^−2^
**406**	1.05	1.05	10	vinblastine	1.2 × 10^−3^	< 3.7	nt
**407**	3.6 × 10^−1^	1.21	50				

^a^ human epidermoid carcinoma cells; ^b^ african green monkey kidney epithelial cells; ^c^ mouse lymphocytic leukemia cells.

## 6. Conclusions

*Annonaceous* acetogenins are a relatively new class of polyketides isolated from *Annonaceae* species. Much attention has been paid to their unique chemical structures and attractive biological activities, including antitumor activity. To clarify their mode of action, synthesis of fluorescently or photoaffinity-labeled analogues in progress [99,100,101,102,103,104,105,106]. Acetogenins have offered not only a challenging target for total synthesis, but they are also fascinating lead compounds for the development of novel antitumor agents. Acetogenin analogues possibly play an important role in cancer therapy in the near future. 
